# Special Issue: Coffee, Fungi, Mycotoxins, and Climate Change

**DOI:** 10.3390/microorganisms11040941

**Published:** 2023-04-04

**Authors:** Robert Russell Monteith Paterson

**Affiliations:** CEB Centre of Biological Engineering, Gualtar Campus, University of Minho, 4700-057 Braga, Portugal; russell.paterson@deb.uminho.pt

Coffee is very lucrative and enjoyed by many. It is a major cash crop, especially for countries without many alternative sources of income. However, production is threatened by climate change, a phenomenon that is now universally accepted as occurring and represents a major threat to societies and industry worldwide. Paterson and Lima [1] expressed great concern in 2010 about the lack of methods to combat climate change. Thirteen years later, there has been little tangible action to reduce climate change, despite many warm words about what will be done “sometime in the future”.

It is apposite to consider what would be the effect of climate change on coffee, particularly in relation to spoilage fungi and the production of mycotoxins. Fungi already cause considerable damage to coffee and mycotoxins have highly significant consequences on human and animal health. The commodity can be rejected by authorities when the concentrations of ochratoxin A (OTA) are too high.

We were hoping for more than four papers on the subject, but were pleased that the quality was so high of those we did receive. Gratitude is extended to Naresh Magan’s group for contributing two papers [2,3], both of which present novel data. Akbar et al. [2] have already been cited 15 times (Scopus (18 February 2023)), indicating the high relevance of their work. The growth and OTA production of *Aspergillus westerdijkiae* were tested in relation to, for example, water activity, temperature and CO_2_ in media and beans. Interestingly, in green coffee-based media, OTA production was optimum at 0.98–0.95 aw and 30 °C. However, in roasted coffee-based media, very little OTA was produced. In stored green coffee beans, optimum OTA was produced at 0.95–0.97 aw/30 °C.

The objective of Akbar et al. [3] (five Scopus citations ((18 February 2023))) was to examine the effect of treatment of coffee beans with gaseous ozone (O_3_) for the control of ochratoxigenic fungi and OTA contamination during storage. The paper revealed that, inter alia, it is unlikely that fungi and the OTA contamination of stored coffee beans would be controlled even with high O_3_ concentrations under wetter conditions.

Our first review on the topic came from Brazil [4], which already has six citations (Scopus ((18 February 2023)). Brazil produces the most coffee globally and we were especially pleased to receive this paper. Temperatures in coffee-producing municipalities in Brazil have increased by about 0.25 °C per decade and annual precipitation has decreased. Therefore, the coffee sector will face serious challenges in the next few decades and the impacts of climate change directly affect coffee mycobiota. Aflatoxins may become dominant with climate change, promoting greater food insecurity in coffee production. Closer attention by authorities is fundamental to stimulate the displacement of areas currently apt for coffee production, that will deteriorate in the future to novel climate zones with suitable climates. This will ameliorate the scarcity of coffee on the world market in the future.

Adhikari et al. [5] (13 citations Scopus ((18 February 2023))) reviewed how (a) suitable areas for coffee cultivation and (b) the toxigenic fungal taxa belonging to *Aspergillus*, *Penicillium*, and *Fusarium* will be affected due to climate change. Studies predict that suitable coffee cultivation areas could drop by 50%. Increased temperatures will see an overall increase in mycotoxin production such as aflatoxins, particularly by *A. flavus*, which grows at higher temperatures. Information regarding climate change parameters and mycotoxin concentrations in real coffee samples is provided. Modelling of future changes in coffee cultivation is also required. Indications show that climate change will result in an increase in mycotoxin contamination.

The current author expresses his appreciation to all the authors of this Special Issue and trusts the papers will be of considerable use to workers in the field. He also hopes that very large advances in reducing climate change will occur in the next 13 years, unlike the previous 13.
